# Virtual‐Based Prenatal Care Methods and Their Reported Outcomes—A Scoping Review

**DOI:** 10.1002/hsr2.71150

**Published:** 2025-08-18

**Authors:** Hamideh Sabetrohani, Jalil Koohpayehzadeh, Abbas Sheikhtaheri, Shahrbanoo Goli, Maryam Biglari Abhari, Afsaneh Keramat

**Affiliations:** ^1^ Student Research Committee, School of Nursing and Midwifery Shahroud University of Medical Sciences Shahroud Semnan Province Iran; ^2^ Preventive Medicine and Public Health Research Center, Community and Family Medicine Department, School of Medicine Iran University of Medical Sciences Tehran Iran; ^3^ Health Management and Economics Research Center, Health Management Research Institute Iran University of Medical Sciences Tehran Iran; ^4^ Department of Health Information Management, School of Health Management and Information Sciences Iran University of Medical Sciences Tehran Iran; ^5^ Digital Health and Artificial Intelligence in Medicine Research Unit, School of Health Management and Information Sciences Iran University of Medical Sciences Tehran Iran; ^6^ Department of Epidemiology, School of Public Health Shahroud University of Medical Sciences Shahroud Semnan Province Iran; ^7^ Community Medicine, School of Medicine Alborz University of Medical Sciences Karaj Iran; ^8^ Center for Health Related Social and Behavioral Sciences Research Shahroud University of Medical Sciences Shahroud Semnan Province Iran

**Keywords:** digital health, mobile health, telehealth, telemedicine, virtual care, virtual prenatal care

## Abstract

**Background and Objective:**

The use of virtual technologies in prenatal care has significantly increased, particularly during the COVID‐19 pandemic; however, the implications of this approach remain a topic of discussion. This review aimed to categorize virtual‐based prenatal care methods and their reported clinical and nonclinical outcomes.

**Methods:**

This scoping review was conducted by searching the Web of Science, PubMed, Scopus, ProQuest, SID, Irandoc, Magiran databases, and Google Scholar search engine from January 2005 to February 2021 and completed until December 2023. Our included studies were quantitative and review studies in English that mentioned virtual prenatal care and related outcomes. We followed the narrative approach for presenting and synthesizing results and PRISMA‐ScR guidelines for the accompanying explanation.

**Results:**

After retrieving 1324 studies and removing duplicates, 35 articles were reviewed. We divided virtual‐based prenatal care into two main categories: only using virtual methods and modified care models by virtual methods. Mhealth was the most widely used virtual care method due to its accessibility to most mothers, low cost, and use of dedicated apps. The reported outcomes were also classified into seven subcategories. Maternal and neonatal outcomes, maternal and provider satisfaction, and change in patient knowledge, attitude, and practice were the three most commonly reported outcomes.

**Conclusion:**

Improvement of a variety of clinical and nonclinical outcomes is anticipated to facilitate the effective implementation of tailored virtual interventions for mothers, ultimately improving health outcomes for both mothers and fetuses.

## Summary

**What is known?:**

- The COVID‐19 pandemic provided the basis for virtual prenatal care development.
- The consequences of using virtual prenatal care need further analysis.

**What is new?:**

- In this review, each virtual modality's reported virtual‐based prenatal care outcomes were depicted separately.
- We classified virtual‐based prenatal care methods into two general categories and then reported their outcomes in seven subcategories.

**What are the clinical implications of this study?:**

- Policies should be directed towards equitable access to new virtual technologies for all.
- Virtual methods should be localized and promoted for each target population.

**Abbreviations**
ANC: antenatal care
FGR: fetal growth restriction
KAP: knowledge, attitude, and practice
PCC: Means Participant, Concept, and Context, a framework presented by the Joanna Briggs Institute for Scope Review [29]
PRISMA‐ScR: Preferred Reporting Items for Systematic reviews and Meta‐Analyses extension for Scoping Reviews Checklist [24]
SID: Scientific Information Database (A native scientific database)
WHO: World Health Organization

## 1 Background

Prenatal care is an essential preventive service that includes 12–14 in‐person visits throughout pregnancy to improve mother and child outcomes based on clinical guidelines developed many years ago. However, evidence‐based studies showed that in‐person services can be provided in fewer numbers and replaced by various virtual modalities in a flexible framework [1]. This issue has received increased attention, especially since the start of the pandemic in the world, although the World Health Organization (WHO) 2020 operational guidance on this matter emphasized that all essential elements of antenatal care (ANC) should be maintained [2].

A study by Clark et al. (2019) on reducing traditional in‐person visits during pregnancy stated that “technology‐based communication and remote monitoring offer advantages for patients and clinicians [3].” In this regard, Peahl et al. (2022) proposed a tailored model for prenatal care that included a combination of in‐person visits, telemedicine in routine care, and consideration of individuals' psychosocial conditions [4].

“Virtual maternity care” is not new [5]. According to the University of Utah's introduction of virtual care, it is a convenient approach to bringing a doctor's appointment to patient's home or workplace using a smartphone, computer, tablet camera, or mobile apps [6]. Wu et al.'s (2020) study showed that mothers can enter into a private online connection with their doctors or health providers to receive care, education, and counseling services [7].

Weigel et al. (2020) showed that maternal satisfaction with prenatal care improved or remained unchanged with virtual methods [8]. Quinn et al. (2021) also indicated high satisfaction levels among mothers with virtual care [9]. Both patients and providers in a new model introduced during the pandemic by Peahl et al. (2021) reported positive experiences with improved access and perceived quality and increased safety for low‐risk mothers during virtual visits, further enhanced maternal satisfaction through better counseling [10]. However, the results of the cross‐sectional study by Futterman et al. (2021) did not show a statistically significant difference between the satisfaction scores of mothers with in‐person and telehealth visits [11].

Several reviews have addressed the issue of virtual prenatal care because there are still many unknown dimensions. These studies have addressed the topic from different aspects. The previous reviews focused on: describing a plan (model) [12] or method about telemedicine [13], telehealth [14], or other virtual methods, available solutions about mobile apps for prenatal care [15], reporting pregnancy‐related outcomes [16], virtual methods interventions [17], reporting impact [18] or effectiveness [19] of virtual care. We used a scoping review because of the broader scope of the questions [20] we addressed in this review and the possibility of summarizing heterogeneous study methods or disciplinary findings [21].

In this scoping review, we sought to capture widely used virtual‐based care along with reported outcomes in a single framework to provide a comprehensive view future national intervention planning.

This review aimed to address the following questions: 1‐ How can we categorize the prevalent methods used in virtual prenatal care? 2‐ What are each method's reported clinical and nonclinical outcomes?

## 2 Methods

To conduct this scoping review, we used the methodology provided by the Joanna Briggs Institute [22, 23] and followed the PRISMA‐ScR 2018 guideline extension for the scoping reviews [21, 24]. (The completed PRISMA‐ScR checklist of this article is available in the Additional Information section under the title “Final version completed‐PRISMA‐ScR.”). Also, we used a scoping approach to conduct this review because of summarizing a wide range of included studies and heterogeneous methodology mapping [25].

### 2.1 Definitions

*2.1.1. Virtual care* is defined as follows: “Any interaction between patients and members of their care team occurring remotely, using technology with the aim of facilitating or maximizing the quality and effectiveness of patient care. Virtual care is simply the modality used to connect and provide care. It can be used for the purposes of assessment, intervention, consultation, education and supervision [26].” In the present review, mHealth, virtual visits, telemedicine, telehealth, eHealth, and smart devices for providing prenatal care were considered virtual care.

*2.1.2*. Most pregnancies are assessed as low‐risk, and neither maternal nor fetal factors contribute to an elevated risk of complications [27]. Nonetheless, some women encounter health‐related complications that impact either their health or that of their infant. These women undergo what is classified as a high‐risk pregnancy [28].

### 2.2 Eligibility Criteria

Quantitative and review studies about virtual‐based care during pregnancy published in journals or conference proceedings were included based on the research questions. Study protocols, papers reported complicated pregnancies or non‐pregnancy outcomes (including weight loss outcomes), studies that only reported descriptions for virtual technology, and nonquantitative methodological designs such as qualitative, commentary, and point of view were excluded. The inclusion and exclusion criteria, including the Participant, Concept, and Context (PCC) Framework, presented by the Joanna Briggs Institute for Scope Review [29], are shown in Table 1.

**Table 1:** Eligibility criteria of this review based on Participant, Concept, and Context (PCC) Framework [29].

Attribute	Inclusion criteria	Exclusion criteria
Participants/Population	Mothers who receiving prenatal care	Women who receiving other services
Concept	Applying virtual methods in prenatal care process or using modified integrated prenatal care models by using a digital technology	Applying virtual methods for women health care not related to pregnancy
Intervention	Different virtual‐based prenatal care (mHealth, telehealth, telemedicine, virtual care/visits, smart devices, eHealth)	Non‐virtual‐based prenatal care
Context	Countries with different level of socioeconomic status	—
Outcome	Reported clinical and nonclinical pregnancy outcomes	Other non‐pregnancy outcomes such as weight loss or physical activity
Type of studies	All of quantitative, review studies and the related conference papers	Other studies such as qualitative, commentary, and point of view
Time period	January 2005 to December 2023	Before 2005
Language	English Language	Other languages

### 2.3 Information Sources

Electronic databases including Web of Sciences, Scopus, PubMed, and ProQuest, as well as the Google Scholar search engine and, the three Persian databases; SID, Irandoc, and Magiran (three native scientific databases), were searched from January 2005 to February 30, 2021. The search were updated from March 1, 2021 to December 30, 2023. Therefore, in total, our information sources included the years 2005 to 2023.

### 2.4 Search

Initially, (HS) performed a pilot search using the primary keywords “telemedicine” and “prenatal care” with the assistance of two distinct methodologists. Other keywords and index terms were extracted from previous studies reference lists. The search strategy included two parts:

– [Women*/Prenatal care [Mesh] OR Antenatal care OR Obstetric care OR Maternal care]
– [Telemedicine [Mesh] OR Tele‐medicine OR Mobile Health OR mHealth OR m‐Health OR Telehealth OR Tele‐health OR ehealth OR e‐Health OR Remote Consultation [Mesh] OR Teleconsultation OR Tele‐consultation OR Telecare OR Tele‐care OR Remote Care OR Tele monitoring OR Tele‐monitoring OR Digital Health OR Virtual Approach OR App OR Digital app OR Mobile app OR Virtual Care OR Virtual Approach].

The “AND” operator was used to combine for the results of these two parts. The final search strategy can be found in Appendix 1.

### 2.5 Selection of Sources of Evidence

The duplicates were automatically removed using EndNote‐X9. In addition to find duplicate records, (HS) manually examined them. Two reviewers (HS and MBA) independently screened the title and abstract of the studies. They evaluated the full texts for eligibility. The team members discussed about any discrepancies to achieve a consensus.

### 2.6 Data Charting Process

Characteristics of the included studies were tabulated using Microsoft Excel in the data extraction table by (HS). Then (AK and AS) inspected the extracted data independently and implemented the necessary corrections. Another reviewer (SG) rechecked the final included studies. Any disagreements were discussed and resolved between them.

### 2.7 Data Items

The main characteristics recorded in the table were: first author, year of publication, study location, purpose, study design, target population, sample size or included studies (for reviews), type of virtual methods, virtual characteristics, reported outcomes, and key findings. Our data extraction tables are depicted in Appendix 2.

### 2.8 Synthesis of Results

Using the Economic and Social Research Council (ESRC) methods program guidelines [30], we used a narrative approach for synthesizing the data. All the entered studies were systematically described and tabulated according to the narrative approach.

## 3 Results

### 3.1 Selection of Sources of Evidence

**Figure 1:**
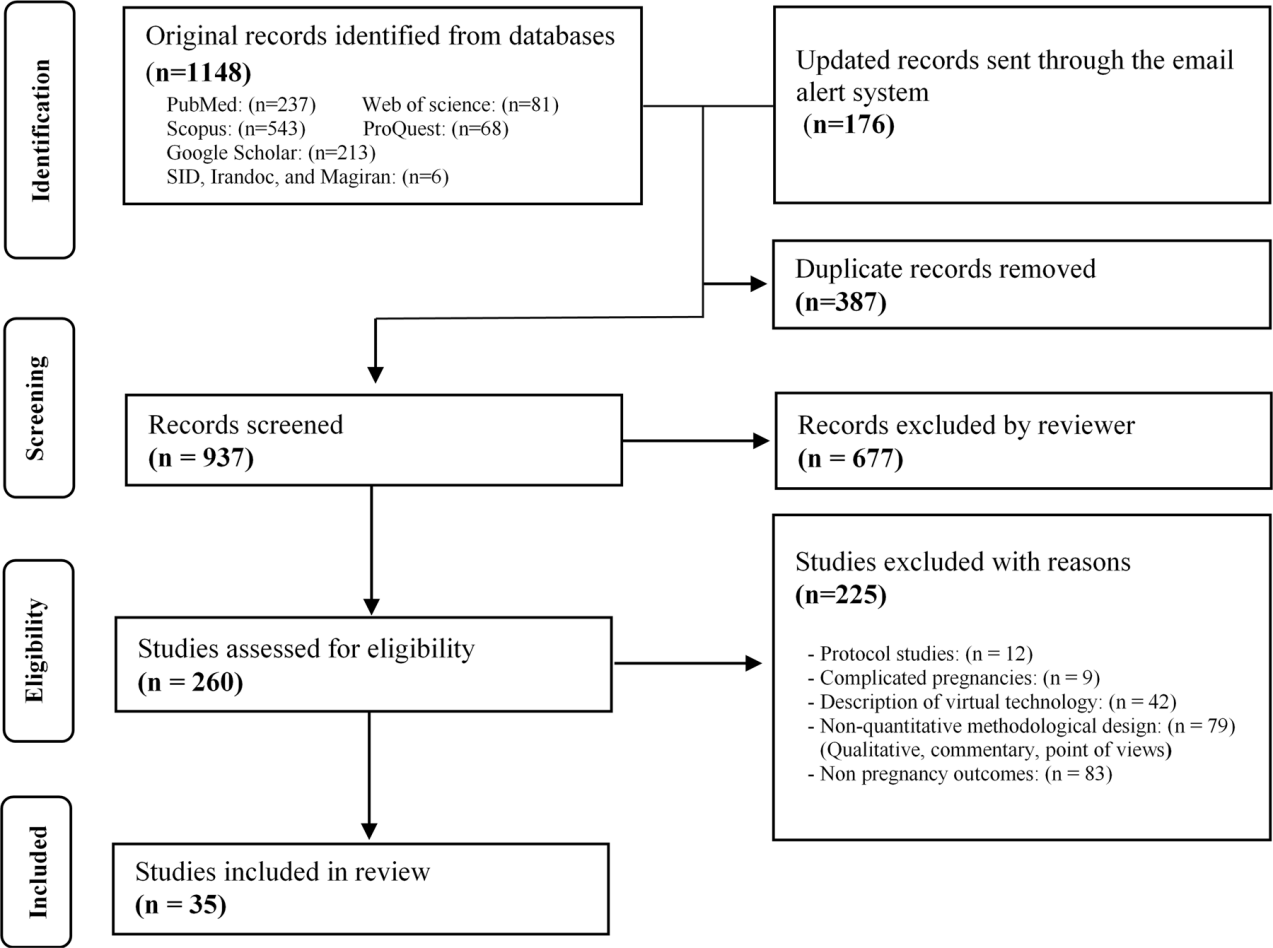
PRISMA flow diagram29 for study selection.

Initially, 1324 articles (1148 original records plus 176 updated records) were reviewed. After removing 387 duplicates, 937 titles and abstracts were evaluated. The full text of 260 articles was assessed in terms of eligibility, and finally, 35 articles were included in the review. The PRISMA flow diagram [29] illustrates our study selection in Figure 1.

### 3.2 Characteristics of Sources of Evidence

The characteristics of included studies are tabulated in Appendix 2. The included studies consisted of (35) quantitative and review studies as below: different methods of a clinical trial (8) [31, 32, 33, 34, 35, 36, 37, 38], cross‐sectional (6) [39, 40, 41, 42, 43, 44], retrospective (3) [45, 46, 47], pilot study (3) [48, 49, 50], evaluation (2) [10, 51], cohort (2) [52, 53], time series analysis (2) [54, 55], secondary data analysis (2) [56, 57], systematic review (2) [12, 58], case‐control (1) [59], follow‐up study (1) [60], pre‐post intervention (1) [61], narrative reviews (1) [62] and overview of literature (1) [63].

### 3.3 Results of Individual Sources of Evidence

**Figure 2:**
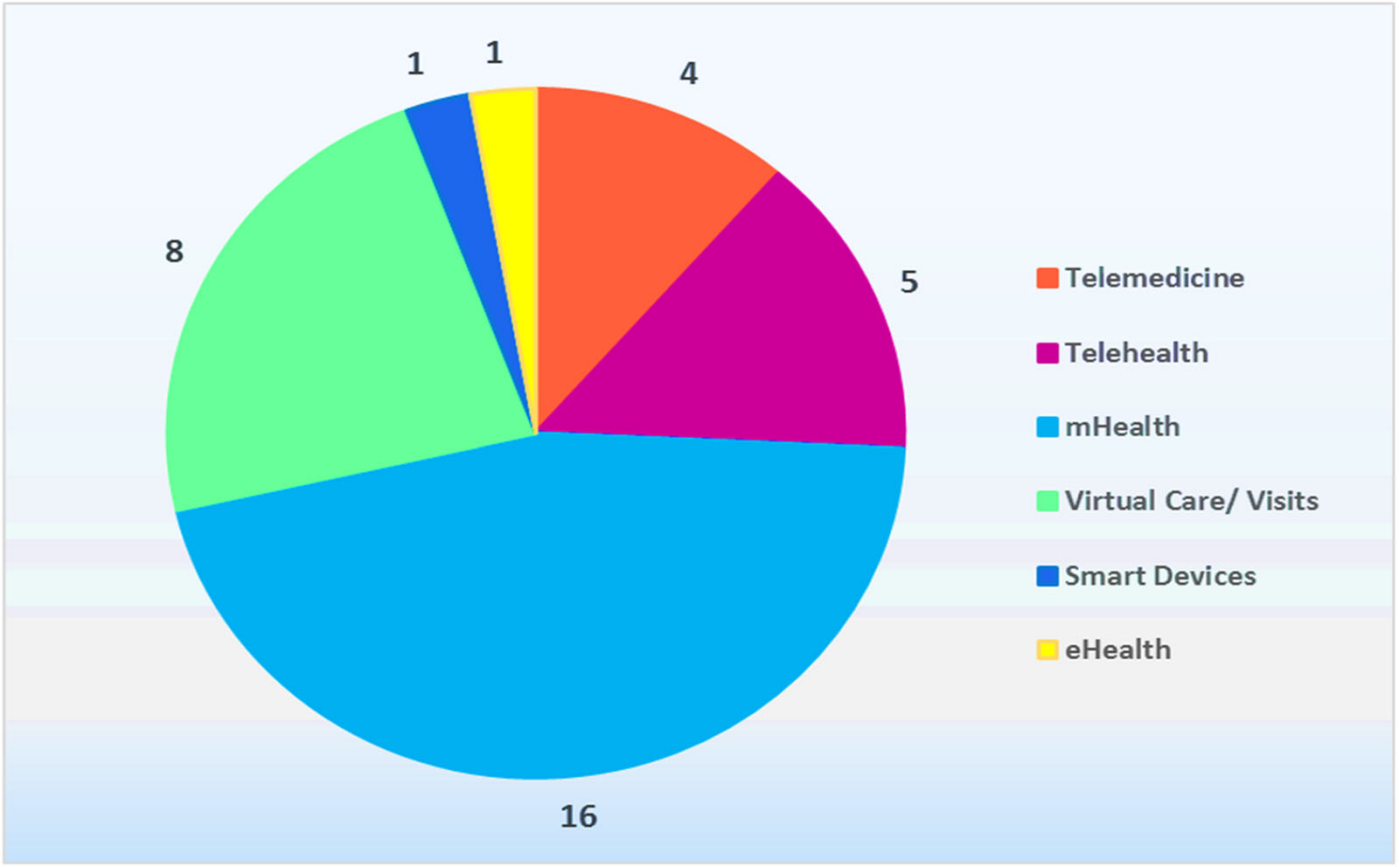
Frequency of virtual‐based methods in included studies.

Out of the 35 studies, mHealth (*n* = 16) was the most commonly used virtual method reported. It was followed by virtual care or visit (*n* = 8), Telehealth (*n* = 5), and Telemedicine (*n* = 4). Each other method has been reported once, including e‐health and smart devices, as illustrated in Figure 2.

**Figure 3:**
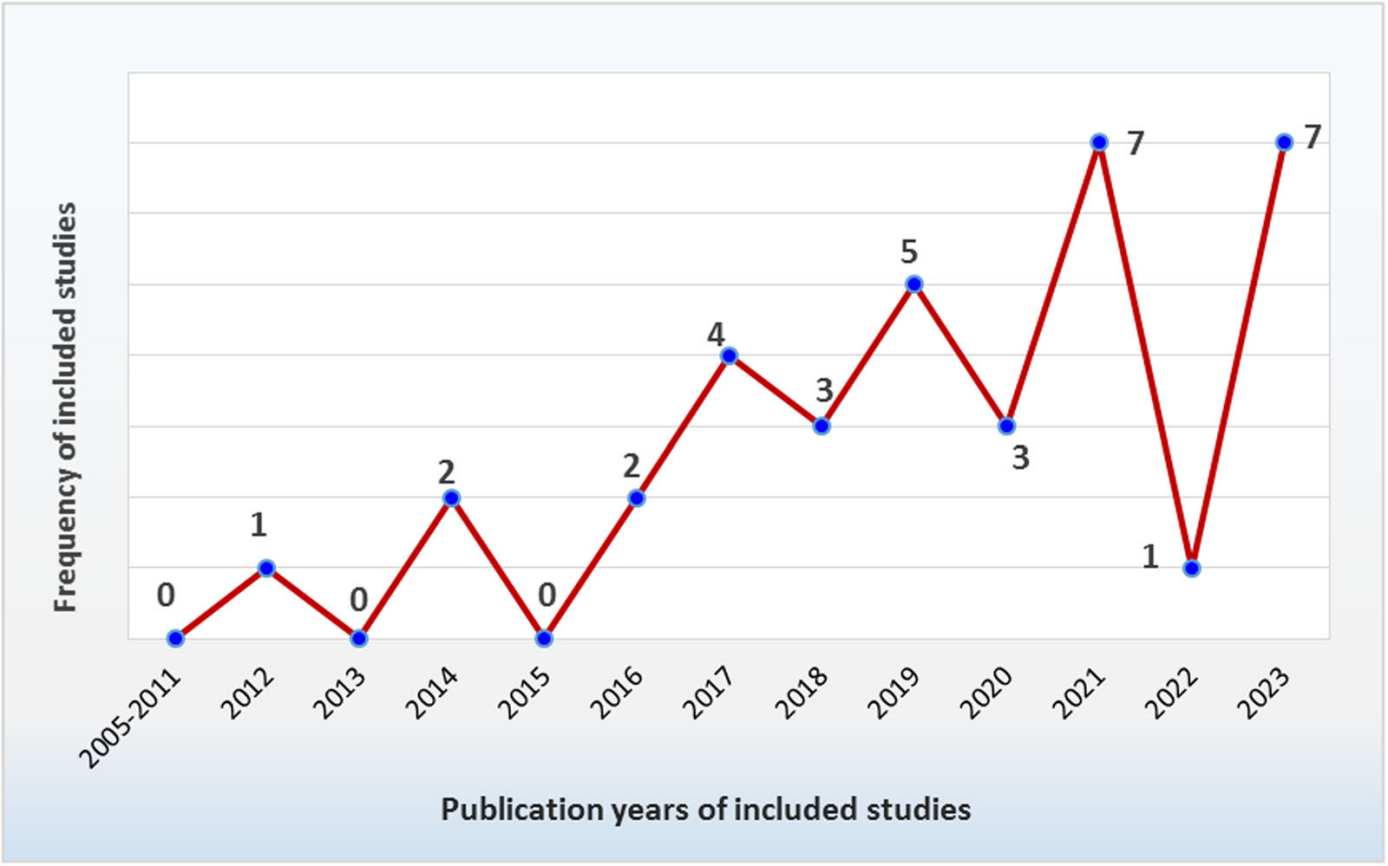
Distribution of included studies by publication year.

The included studies were all written in English, moreover, most were published in 2021 and 2023, as shown in Figure 3.

**Figure 4:**
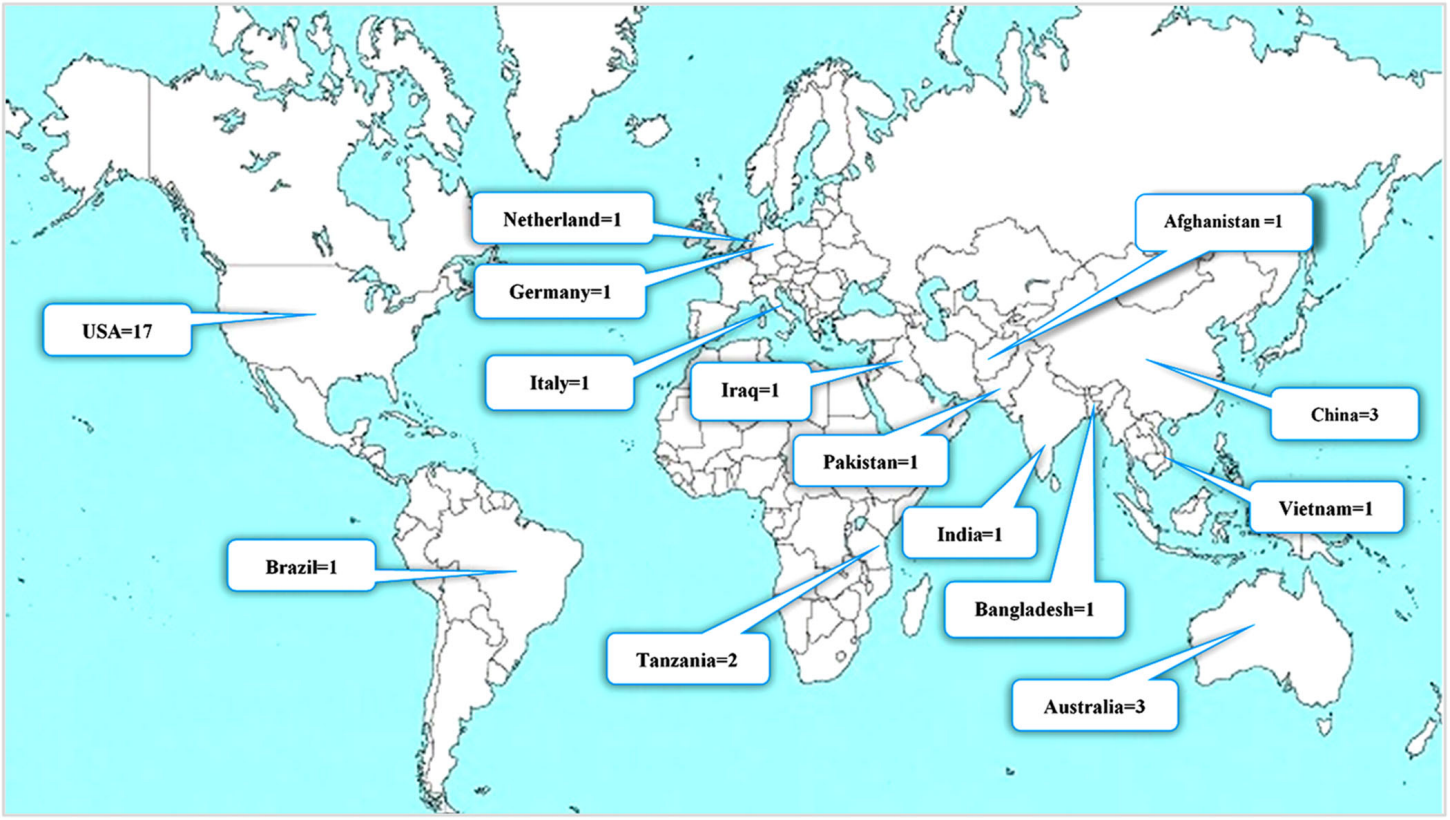
Included studies distribution based on study location. *Note: We used the Pxfuel website map [64] *image to display the frequency of included studies based on geographical locations*.

Almost, half of the included studies have been carried out in the United States, and others in 13 various countries, as mapped in Figure 4.

### 3.4 Synthesis of Results

In the present scoping review, we synthesized and reported our included studies based on virtual‐based methods and their reported outcomes in prenatal care classifications.

#### 3.4.1 The Classification of Included Studies by Virtual‐Based Methods

Different virtual care methods in prenatal care can be divided into two general categories: 1‐ only using virtual methods (*n* = 22) [31, 32, 34, 35, 36, 37, 38, 39, 40, 41, 43, 45, 46, 47, 48, 49, 50, 57, 58, 60, 61, 63] and 2‐ modified care models by virtual methods (*n* = 13) [10, 12, 33, 42, 44, 51, 52, 53, 54, 55, 56, 59, 62].

Studies with the title “only using virtual methods” include studies in which only a virtual method was introduced (to present the results of an intervention or review) and did not present any modified pregnancy care schedule/model. On the other hand, studies titled “Modified care models by virtual methods” include studies in which a new care model is presented using virtual methods, or a modified program with virtual methods is introduced.

##### 3.4.1.1 Only Using Virtual Methods

According to our review, among the 22 included studies in this sub‐section, mHealth (*n* = 16) [31, 32, 34, 35, 36, 37, 38, 45, 46, 47, 48, 49, 50, 58, 60, 61] was the most prevalent virtual method for prenatal care, employing different modes (text messages, voice, and apps) through mobile phones. The most common form of mHealth used in prenatal care was: various apps (*n* = 8) [34, 38, 45, 46, 48, 50, 58, 61], sending text messages with a phone or apps (*n* = 6) [31, 35, 36, 37, 49, 60], and voice messages alone or in combination with text (*n* = 2) [32, 47]. The most important goals of these options were to provide information about receiving services, increase awareness, modify attitudes, and promote healthy behaviors of mothers.

The second method of virtual care or visit was telemedicine (*n* = 2) [41, 57], which reflected the benefits and facilities of virtual methods through video conferencing, visiting or forming virtual groups for follow‐up, training and counseling expectant mothers. Telehealth [39], virtual care or visits [40], e‐health [63], and smart devices [43] were other methods by only one included study from each technique. These studies showed applying virtual methods for interpreting ultrasound and test results, virtual training, counseling, and self‐monitoring.

##### 3.4.1.2 Modified Care Models by Virtual Methods

This sub‐section contained 13 of our included studies with three methods. These virtual methods in the adjusted care models included virtual care or visits (*n* = 7) [10, 33, 42, 44, 51, 53, 56], telehealth (*n* = 4) [54, 55, 59, 62], and telemedicine (*n* = 2) [12, 52]. The bases of these models were: (1) Reducing the doctor or midwife's in‐person visits compared to traditional care. (2) Turning part of the mothers' visit into a remote visit, mainly through virtual methods based on high‐risk or low‐risk pregnancies. (3) Self‐monitoring at home with delivery of electronic devices (fetal heart rate or blood pressure monitoring). (4) Online counseling services and providing required training packages, and (5) taking part in online groups to exchange experiences and support mothers.

#### 3.4.2 The Classification of Included Studies by Their Reported Clinical and Nonclinical Pregnancy Outcomes

All the included studies referred to one or more clinical or nonclinical pregnancy outcomes. In this section, these outcomes are listed in order based on the abundance of the included studies:

##### 3.4.2.1 Maternal‐Neonatal Outcomes

Maternal‐neonatal outcomes have been mentioned in 11 included studies [12, 33, 51, 52, 53, 54, 55, 59, 62], of which two [45, 46] were in the category of only virtual methods, and nine [12, 33, 51, 52, 53, 54, 55, 59, 62] were in the category of modified care models by virtual methods. Two studies [46, 53] mentioned composite outcomes [Preterm birth, low birth weight, birth defects, stillbirth, and neonatal asphyxia (umbilical cord blood pH less than 7.0) placental abruption, and neonatal intensive care unit] and two studies [52, 55] investigated primary and secondary outcomes.

Maternal‐neonatal outcomes were reported in seven studies [12, 46, 52, 53, 55, 59, 62] using only the virtual method or the use of the model, with no change or difference compared to usual care. Meanwhile, the above four studies [46, 52, 53, 55] were also statistically significant. In the “OB Nest” [33] and “OB CareConnect” [51] models compared to the traditional group, except for gestational diabetes and pre‐eclampsia diagnosis, which had a statistically significant increase in the model group, there was no difference in maternal outcomes. In another study [54], the number of women diagnosed with gestational diabetes was significantly higher, but in the detection of pre‐eclampsia and fetal growth restriction (FGR), missed FGR, and rates of perinatal mortality reported no change. Educational curriculum based on a mobile phone [45] also reported a significant reduction in the risk of some maternal‐neonatal outcomes (Such as risk of gestational diabetes, induced abortion, postpartum infection, fetal intrauterine distress, and neonatal malformation).

##### 3.4.2.2 Patient's and Provider's Satisfaction

Of the included studies, 11 studies [10, 33, 34, 40, 42, 48, 49, 51, 61, 62, 63] referred to satisfaction. Six studies [34, 40, 48, 49, 61, 63] were in the only virtual method category and the rest [10, 33, 42, 44, 62] were in modified care models by virtual methods. A study [40] showed that virtual‐based prenatal care due to the COVID‐19 pandemic was associated with lower patient satisfaction, and another study [34] reported no change in satisfaction between Babyscripts app users and the traditional group.

Meanwhile, the four included studies [33, 61, 62, 63] have reported higher levels of satisfaction among patients and providers, which was statistically significant in a method [61] and a model [33]. A study [44] has also presented a substantial increase in the satisfaction score in the combined use of virtual and in‐clinic prenatal care. The four included studies [10, 42, 48, 49] also mentioned mothers' and providers' high and very high agreement on the satisfaction scale (of at least 59.8%–99%) in virtual methods [48, 49] or models [10, 42] with routine care.

##### 3.4.2.3 Patient's Knowledge, Attitude, and Practice Changes

In 10 included studies [31, 32, 35, 36, 38, 39, 47, 50, 58, 60], mothers' knowledge, attitude, and practice (KAP) were reported, all related to the only using virtual methods sub‐section. In a study [39], the attitude of mothers towards using telehealth to receive counseling was high ( > 75%). In all the included studies related to mothers' use of mHealth [31, 32, 35, 36, 38, 47, 50, 58, 60], an increase in mothers' knowledge and awareness (*n* = 4) [31, 35, 47, 60], improvement of their attitudes and beliefs (*n* = 2) [38, 50], and improvement of their health performance or behaviors (*n* = 3) [32, 36, 58] have been reported.

Also, the reported scores of three studies [31, 35, 60] on increasing knowledge and awareness, two [38, 50] on improving attitudes and beliefs, and two [32, 36] on improving mothers' performance or health behaviors were statistically significant.

##### 3.4.2.4 Frequency of Virtual Services

Nine included studies [34, 36, 37, 40, 42, 49, 57, 58, 59] addressed the issue of the amount of prenatal care with different virtual methods [34, 36, 37, 40, 49, 57, 58] or modified care models [42, 59]. Two studies [34, 57] specifically mentioned the decreased mothers' in‐person visits, one of which was significant. Also, three studies [40, 42, 59] listed the change and improvement of virtual services compared to in‐person ones, two of which were significant. Finally, four studies [36, 37, 49, 58] also reported a relative improvement in the total number of antenatal cares, which was significant in two cases.

##### 3.4.2.5 Direct and Indirect Cost and Time

Regarding time and costs associated with prenatal care, we included six studies [37, 42, 48, 56, 57, 62], three from each only virtual method [37, 48, 57] and modified models [42, 56, 62]. Among these, four studies [37, 42, 48, 57] each mentioned the adequacy and improvement of the time spent on care. In addition, one study [42] pointed to decreased time for travel and less time off from work and childcare.

Regarding costs, two studies [57, 62] pointed to a reduction (significant for one of them [57]) in the overall cost of care. Another study [56], using the “OB Nest” model, reported that despite the decrease in travel and overhead costs, the cost of nursing has increased.

##### 3.4.2.6 The Quality of Care, Access and Safety

Five of our studies [10, 33, 37, 41, 61] dealt with these outcomes (three related to only methods [37, 41, 61] and two among modified models [10, 33]). In one of them [41], mothers had significantly greater agreeability that they could see and hear their provider with telemedicine, and it was also easier for them to see doctors or specialists with telehealth. In another study [10], most patients and almost all providers (In low‐risk pregnancy and with the delivery of self‐care devices) reported that virtual visits improved access to care and safety.

While a study [37] mentioned improving the quality of antenatal care, another one [61] using the “CommCare app” reported a significant increase in the quality score related to health counseling, technical services provided, and health education quality. Also, according to another study [33], reported “OB Nest” care, there was no statistical difference in perceived quality of care.

##### 3.4.2.7 Patient's and Provider's Attitude and Preferences Toward Virtual‐Based Methods

All four included studies [40, 41, 42, 43] related to the subject belong to the “only using virtual methods” sub‐section. There were conflicting views and preferences in this regard. Although one study [41] reflected that most patients sought telemedicine as a choice for future visits, two other studies reported differently. One study [43] pointed to a skeptical perspective toward pregnant mothers' self‐monitoring, and the second study [40] stated that 89.9% of mothers preferred in‐person visits in non‐pandemic conditions. The latest study [42] also reported that most mothers desired to combine in‐person and virtual prenatal visits.

The classification of virtual‐based methods (only using virtual methods or modified care models by virtual methods) in prenatal care and their reported outcomes is presented in Tables 2 and 3.

**Table 2:** Summary classification of virtual‐based methods (only using virtual methods) in prenatal care and their reported outcomes from included studies.

	Virtual methods	Specific apps	First author‐year	Reported clinical and nonclinical outcomes	Reported changes	Conditions
Only using virtual methods	Telemedicine		Tozour [41]–2021	(1)‐ The patient's/provider's digital experience	Higher scores	Significantly
				(2)‐ Patients'/provider's desire for future use:	Trend to future use	*—*
				‐ Able to see and hear their provider	Greater agreeability	Significantly
				‐ TM visits were as good as in‐person one	Greater agreeability	Significantly
				‐ Easier to see doctors or specialists	Greater agreeability	Significantly
			Barbour [57]–2017	(1)‐ In‐clinic prenatal visits	Decrease	Significantly
				(2)‐ Time usage for the patient	Decrease (3 h totally)	Significantly
				(3)‐ Visit‐related costs	Decrease	Significantly
	Telehealth		Cheung [39]–2023	(1)‐ Using for routine prenatal checkups	Lower scores ( < 20%)	*—*
				(2)‐ Addressing pregnancy‐related concerns	Lower scores ( < 20%)	*—*
				(3)‐ Using for prenatal education talks, prenatal and postpartum exercise, and addressing breastfeeding problems.	Higher scores ( > 75%)	
				(4)‐ Explaining pregnancy exam results, medical history‐taking, self‐monitoring blood pressure	Medium scores	*—*
	mHealth	*mMoM intervention*	Dao [31]–2023	(1)‐ Awareness about the danger signs of pregnancy and the nutritional supplements.	Improve	Significantly
				(2)‐ Maternal health‐related knowledge and care‐seeking	Improve	Significantly
		*The PUMCH curriculum*	Hao [45]–2023	(1)‐ Pregnancy outcomes (Risk of gestational diabetes, induced abortion, postpartum infection, fetal intrauterine distress, and neonatal malformation)	Reduction risk	Significantly
				(2)‐ Pregnancy outcomes (Premature rupture of membranes and small for gestational age)	Reduction risk	*—*
				(By pregnancy psychology and pregnancy nutrition topics)		*—*
		*Different MCH Apps*	Zhang [46]–2022	‐ Composite adverse pregnancy outcome (CAPO)	No difference for incidence of	*—*
				(Preterm birth, low birth weight, birth defects, stillbirth, and neonatal asphyxia)	CAPO	*—*
		*mMitra*	Murthy [32]–2020	‐ Impact on maternal care practices, knowledge and health outcome of Anemia	Increase (In some indicators)	Significantly
		*MAMA program*	Lebrun [60]–2020	‐ Correct mother's knowledge about MNCH	Increase	Significantly
		*Babyscripts*	Marko [34]–2019	(1)‐ Average number of in‐person OB visits	Decrease	*—*
				(2)‐ Satisfaction (patients & providers)	No change	*—*
			Masoi [35]–2019	(1)‐ Mother's knowledge about danger signs	Increase	Significantly
				(2)‐ Birth preparedness	Increase	Significantly
		*Aponjon*	Chowdhury [47]– 2019	‐ Knowledge and positive behaviors for MNH	Increase	*—*
		*PANDA*	Borsari [48]–2018	(1)‐ Considered the time of the visit	Adequate	*—*
				(2)‐ Satisfaction	91.9% ‘very satisfied’	*—*
			Alhaidari [49]–2018	(1)‐ Median number of visits	Improve 2 to 4	Significantly
				(2)‐ Satisfaction (agree/strongly agree)	Range 59.8%– 90.7%	*—*
		*PRENACEL*	Oliveira‐Ciabati [36]–2017	(1)‐ ≥ 6 ANC visits	Increase	Significantly
				(2)‐ Rates of syphilis/HIV's testing during ANC	Increase	Significantly
		*CommCare*	McNabb [61]–2016	(1)‐ Quality score	Increase	Significantly
				(2)‐ Satisfaction	Increase	Significantly
				(3)‐ Health counseling	Improve	Significantly
			Lund [37]–2014	(1)‐ ≥ 4 ANC visits	Improve 31% to 44%	*—*
				(2)‐ Timing and quality of ANC	Improve	*—*
		*Text4baby*	Evans [38]–2014	(1)‐ Belief to risks of alcohol during pregnancy	Increase agreement	Significantly
				(2)‐ Belief to importance of taking vitamins	Increase agreement	Significantly
		*Text4baby*	Evans [50]–2012	(1)‐ Attitude to “prepared to be a new mother”	Increase agreement	Significantly
				(2)‐ Attitude to “alcohol will harm to my baby’”	Increase agreement	Significantly
			Feroz [58]–2017 (Review)	(1)‐ Changing the behavior of pregnant	Effective	*—*
				(2)‐ Antenatal care services	Improve	*—*
	Virtual care/visits		Liu [40]–2021	(1)‐ Change in‐person to virtual visits	81.3%	*—*
				(2)‐ Association sociodemographic variables (Pandemic duration, Stress, ANC Changes)	Decrease (less satisfied)	Significantly
				(3)‐ Preference in‐person ANC after pandemic	89.9%	*—*
				(4)‐ Satisfaction rate by preference (virtual vs in‐person)	Decrease	Significantly
	Smart devices		Schramm [43]–2019	(1)‐ Selection e‐device to before seeing a doctor in fewer baby movements	Majority	Significantly
				(2)‐ Attitude toward the use of eHealth with several emergency visits vs without one	No change	*—*
	eHealth		Van Den Heuvel [63]–2018 (Review)	(1)‐ Remote monitoring and counseling.	*—*	*—*
				(2)‐ Satisfaction/Comfort	High level	*—*
				(3)‐ Most searched topics (Fetal development, complications and healthy lifestyle)	*—*	*—*

**Table 3:** Summary classification of virtual‐based methods (modified care models by virtual methods) in prenatal care and their reported outcomes from included studies.

	Virtual methods	Specific models	First author‐year	Reported clinical and nonclinical outcomes	Reported changes	Conditions
*Modified care models by virtual* methods	Virtual care/visits	*Coronavirus Disease 2019 Model/Incorporating Model*	Peahl [10]–2021	(1)‐ Access to care	Improved as follows: (68.8% ‐to‐ 96.1%)	*—*
				(2)‐ Believed safe care	(53.3%‐ to‐ 62.1%)	*—*
				(3)‐ Satisfaction	(77.5%‐ to ‐83.1%)	*—*
		*OB Nest Model*	Theiler [56]–2021	(1)‐ Nursing cost	Increase	*—*
				(2)‐ Travel & overhead cost	Decrease	*—*
		*Audio‐Only Virtual Prenatal Visits*	Duryea [53]–2021	‐ Composite outcome (placental abruption, stillbirth, neonatal intensive care unit, and umbilical cord blood pH less than 7.0)	No change	Significantly
		*Audio‐Only Virtual Prenatal Visits*	Holcomb [42]–2020	(1)‐Virtual prenatal visits as scheduled vs in‐person	88% vs. 82%	Significantly
				(2)‐ Satisfaction (needs were met)	99%	*—*
				(3)‐Need to transportation, job's time away & childcare	Decrease	*—*
		*OB Nest Model*	Tobah [33]–2019	(1)‐ Pregnancy‐related stress at 14 and 36 weeks	Lower	Significantly
				(2)‐ Maternal complications (outcome)	No change (Except GDM)	Significantly
				(3)‐ Quality of care	No change	Significantly
				(4)‐ Satisfaction	Higher	Significantly
		*OB CareConnect Model*	Pflugeisen [44]–2017	‐Satisfaction	Correlated with cohort	Significantly
		*OB CareConnect Model*	Pflugeisen [51]–2016	(1)‐ Pre‐eclampsia diagnosis	Higher	Significantly
				(2)‐ Other outcome and system use	No change	*—*
	Telemedicine	*Multimodal Prenatal Health Care Model*	Ferrara [52]–2023	(1)‐ Maternal primary outcomes (Pre‐eclampsia and eclampsia, severe morbidity, cesarean delivery and preterm birth)	No differences	Significantly
				**(Exception NICU admission rates)**	Decrease (In T2), Increase (In T3)	*—*
				(2)‐ Maternal secondary outcomes (Gestational hypertension, gestational diabetes, depression, venous thromboembolism, newborn Apgar score, transient tachypnea, and birth weight)	No relevant changes	*—*
		*The Michigan Plan for Appropriate Tailored Healthcare (MiPATH)*	Barrera [12]–2021 (Review)	(1)‐ Health outcomes for patients without medical conditions who received telemedicine visits	No differences	*—*
				(2)‐ Home monitoring:	Feasible	*—*
				‐ Blood pressure & weight	Not assessed	*—*
				‐ Fetal heart tones and fundal height		
	Telehealth	*Antenatal Telehealth Models*	Atkinson [62]–2023	(1)‐ Adverse maternal or neonatal outcomes	No differences	*—*
				‐Rarer outcomes, such as stillbirth or maternal mortality	Increase	*—*
				(2)‐ Cost of care	Decrease	*—*
				(3)‐ Satisfaction	Higher	*—*
		*Reduced Contact Prenatal Care Model*	Mei [59]–2023	(1)‐ Adequate prenatal care (Reduced model VS in‐person)	Higher	Significantly
				(2)‐ Maternal and neonatal outcomes	No differences	*—*
		*Telehealth‐integrated Antenatal Care*	Thirugnanasundralin‐gam [54]–2023	(1)‐ Detection of pre‐eclampsia and fetal growth restriction (FGR), missed FGR, or rates of perinatal mortality.	No differences	Significantly
				(2)‐ The number of women diagnosed with gestational diabetes.	Higher	Significantly
		*Telehealth‐integrated Antenatal Care*	Palmer [55]–2021	(1)‐ Maternal primary outcomes (Fetal growth restriction, pre‐eclampsia, and gestational diabetes)	No change	Significantly
				(2)‐ Maternal secondary outcomes (Stillbirth, neonatal intensive care unit admission, and preterm birth)	No change	Significantly
					(Except reduction in preterm birth in high risk model)	Significantly

## 4 Discussion

### 4.1 Summary of Evidence

In this scoping review, we categorized virtual‐based prenatal care methods into two categories: *only using virtual methods* and *modified care models by virtual methods*. We also summarized clinical and nonclinical reported outcomes in seven sub‐sections: *maternal‐neonatal outcomes, patient and provider satisfaction, patient's knowledge, attitude, and practice changes, frequency of virtual services, direct and indirect cost and time, quality of care, access and safety, patient and provider attitude and preferences toward virtual‐based methods*.

For years, new digital technology as supplementary in counseling and self‐care has been used for mothers with high‐risk pregnancies and reducing perinatal mortality and morbidity in remote areas and rural communities [11]. During the COVID‐19 pandemic, rapidly virtual prenatal care has been developed around the world and has changed traditional care plans [65, 66].

Studies indicate that mobile phones are attractive, cheap, widespread, and have effective technical capabilities for delivering health interventions even in low and middle‐income countries [67, 68]. MHealth interventions during pregnancy have been exploited for limited access to health services, living in remote areas, and inadequately skilled health workers [69]. Mobile phones are innovative solutions with immense potential to overcome barriers to accessing ANC services [70]. Despite the high acceptability level of mHealth especially in rural areas [71], mothers and providers mentioned some restrictions to using mHealth for antenatal care such as discontinuity of care, poor internet connectivity, and lack of trust in technology [72, 73].

On the other hand, some available studies [11, 12, 41] showed that pregnancy care through telemedicine is safe and produces similar results compared to traditional models of care and was considered an important alternative to in‐person consultations [66]. The American College of Obstetricians and Gynecologists (ACOG) has recently encouraged providers to integrate telehealth into prenatal care, emphasizing that this technology can effectively complement the current care standard [11]. Notably, despite some studies reporting no difference in the primary and secondary maternal and neonatal outcomes in telemedicine [12, 52] and telehealth [59, 62] with routine methods, mothers and providers noted challenges in using these platforms. However, challenges were more noticeable in low or middle‐income countries [74].

The results of the virtual visit showed that the experiences of patients and providers were similar and included perceived improved access to care through decreased barriers, perceived high‐quality virtual visits for low‐risk patients, increased safety during the pandemic, and enhanced patient satisfaction from counseling [10, 33, 51]. There were worries that unequal access to virtual visits could heighten maternity care inequities including the lack of home devices affecting care quality and safety (e.g., blood pressure cuffs), dissatisfaction with poor patient‐provider relationships [10], and hesitation in using technology [75].

In recent studies, virtual‐based prenatal care sometimes focused on low‐risk pregnancies [33, 51] and sometimes included both high‐risk and low‐risk groups [54, 55]. However, significant changes in maternal and newborn outcomes have not been reported, except for a few related components (e.g., reduction in preterm birth in high risk model, diagnosis of gestational diabetes).

Studies using the preferences of mothers and providers or pregnancy outcomes have addressed the use of virtual methods or modified models of prenatal care in the coming years and have reported different perspectives. In one study, some mothers expressed their willingness to use the virtual method in the future [73], and another 90% of mothers preferred to use in‐person visits in non‐pandemic conditions [40]. In most of the current studies [10, 42, 54, 75, 76, 77, 78], a combination of virtual and in‐person visits was suggested, which seems to include the advantages of both methods in addition to being more feasible and flexible.

Since it is necessary to pay attention to the context of demographic groups such as socioeconomic status, technological infrastructure, and preferences of mothers and providers [66, 75], policymakers can plan the appropriate combination of methods for their target population using the results of studies on pregnancy outcomes.

### 4.2 Strengths and Limitations

We had some strengths and limitations in our scoping review. The included studies had an appropriate methodological diversity, including almost all quantitative methodologies and at least one statistically reported clinical or nonclinical outcome. In addition, our studies included ranging methods from simple text messages to more advanced technologies. One of the limitations of the present study is that despite the geographical dispersion of the included studies, almost half of them belonged to the United States. This issue can challenge the possibility of using the introduced methods or models or the reported maternal and newborn outcomes in countries with low socioeconomic status. Also, the studies we included were only in English.

## 5 Conclusion

The present study classified virtual‐based prenatal care methods differently and reported their clinical and nonclinical outcomes. Accordingly, mHealth was the most widely used methods, and the most reported outcomes were related to maternal and newborn outcomes, maternal and provider satisfaction, and changes in patient knowledge, attitude, and practice. Despite the various benefits of using virtual methods in prenatal care, the findings of this study indicated that the selected virtual‐based methods in each context should have minimum adverse outcomes. At the same time, paying attention to the barriers and infrastructure required before implementation was necessary. It is advisable to conduct additional research into the specific needs of mothers in each region to facilitate the design and implementation of effective virtual‐based methodologies.

## Author Contributions

**Hamideh Sabetrohani:** writing – original draft, conceptualization, writing – review and editing, visualization, project administration, data curation, resources. **Jalil Koohpayehzadeh:** methodology, validation, data curation, supervision, formal analysis. **Abbas Sheikhtaheri:** investigation, methodology, validation, writing – review and editing, software, data curation, supervision. **Shahrbanoo Goli:** methodology, validation, formal analysis, data curation, supervision. **Maryam Biglari Abhari:** methodology, validation, data curation, resources. **Afsaneh Keramat:** conceptualization, investigation, methodology, validation, writing – review and editing, visualization, project administration, data curation, supervision, formal analysis.

## Disclosure

The lead author Afsaneh Keramat affirms that this manuscript is an honest, accurate, and transparent account of the study being reported; that no important aspects of the study have been omitted; and that any discrepancies from the study as planned (and, if relevant, registered) have been explained.

## Conflicts of Interest

The authors declare no conflicts of interest.

## Transparency Statement

The lead author Afsaneh Keramat affirms that this manuscript is an honest, accurate, and transparent account of the study being reported; that no important aspects of the study have been omitted; and that any discrepancies from the study as planned (and, if relevant, registered) have been explained.

## Supporting information

New appendix 2.

New revised appendix 1.

Final revised completed‐PRISMA‐ScR.

## Data Availability

The data supporting this study's findings are available (Including two appendices and a filled PRISMA checklist). We confirm that the data supporting the findings of this study are available within the article and its supplementary materials. (Including two appendices and a filled PRISMA checklist).
