# Supplementary material for: Virtual‐Based Prenatal Care Methods and Their Reported Outcomes—A Scoping Review
Source: Health Sci Rep. 2025 Aug 18;8(8):e71150. doi: 10.1002/hsr2.71150 (PMC12361639; doi:10.1002/hsr2.71150)
Supplement: Supplementary file 1 — New appendix 2. [file HSR2-8-e71150-s002.pdf]

Appendix 2

Characteristics table of included studies about using virtual-based methods in prenatal care and their reported outcomes

No	First author/ (Year)/ Study location	Purpose	Study design	Target population (with sample size) /Included studies	Different virtual methods/ *(Introduced model or specific app)*	Virtual characteristics	Reported outcomes	Key findings
**1**	Cheung, (2023), China (Hong Kong)	Assess pregnant women's attitudes, concerns, and perceptions regarding telehealth obstetric clinic services	Prospective cross-sectional study	**664** participants distributed across different pregnancy stages	Telehealth	The potential use of telehealth obstetric clinical services in Hong Kong.	Consultations for routine prenatal checkups and certain clinical issues	(1)- less favored for physical complaints and routine care<br>(2)- Desirable option for prenatal educational talks, disclosure of investigation results, postpartum exercise classes, and discussing breastfeeding problems.<br>(3)- Benefits of telehealth clinics (e.g. shorter traveling and waiting times, improved accessibility, and lower costs.)<br>(4)- Concern regarding telehealth clinics (e.g. potential errors or delays in diagnosis, limited interactive engagement with medical professionals, privacy issues linked to network and technical problems.)
**2**	Dao, (2023), Vietnam	Promote antenatal health among pregnant by an SMS-based intervention in a remote region	Cluster randomized control trial	Intervention group (**N=640**) Control group (**N=315**)	mHealth *(mMoM intervention)*	**75** SMS messages delivered between weeks **5** and **42** of pregnancy, two to three times per week	MNCH knowledge, behavior, and self-reported health status of pregnant women in pre-post intervention	(1)- Significant improvements in awareness about the danger signs of pregnancy and the importance of nutritional supplements.<br>(2)- Significant improvements in antenatal care-seeking behaviors and the intake of nutritional supplements.
**3**	Hao, (2023), China	Develop & evaluate the effectiveness of a mobile-based prenatal education in improving pregnancy outcomes.	Retrospective real-world	**1941** participants	mHealth *(The PUMCH mobile prenatal care curriculum)*	**436** courses across 9 topics, 1-2 courses available for each topic daily, and each course required just about 5 minutes to complete.	Compared adverse pregnancy outcomes between the completing group with non-completing	(1)- Significant reduction in the risk of gestational diabetes mellitus, induced abortion, postpartum infection, fetal intrauterine distress, and neonatal malformation in completing group.<br>(2)- Completing courses related to topics of pregnancy psychology and pregnancy nutrition was associated with reduced risks of premature rupture of membranes and small for gestational age infants, respectively.
**4**	Ferrara, (2023), USA	Evaluate a multimodal model of in-office and telemedicine prenatal health care and its association with maternal and newborn health outcomes.	Cohort study	**151 464** individuals at 3 time intervals (**T1**: July 1, 2018-February 29, 2020) (**T2**:March 1, 2020-December 5, 2020) (**T3**:December 6, 2020-October 31, 2021)	Telemedicine *(Multimodal Prenatal Health Care Model)*	A multimodal prenatal health care model with use of both in-office and telemedicine visits during the COVID-19 pandemic	(1)- Primary outcomes (e.g. preeclampsia and eclampsia, severe maternal morbidity)<br>(2)- Secondary outcomes (e.g. gestational hypertension, gestational diabetes)	(1)- Increased mean number of prenatal visits via telemedicine from 11.1% to 21.3% of the total visits per individuals.<br>(2)- No clinically significant differences in prenatal health care process measures.<br>(3)- No clinically significant changes in most of our primary health outcomes.<br>(4)- Slightly increased in Rates of gestational hypertension, GD, and depression from T1 to T2 and from T2 to T3

**5**	Atkinson, (2023), Australia	(1)- Describe the different telehealth technologies available for antenatal care.<br>(2)- Review the telehealth impacts on the risk of adverse pregnancy outcomes.<br>(3)- Health economic analyses	Narrative review	---	Telehealth *(Antenatal Telehealth Models)*	Review two broad types of telehealth in antenatal care: real-time telehealth and remote telehealth	(1)- Clinical safety and health outcomes for the mother or baby (e.g. maternal mortality, serious morbidity, or stillbirth)<br>(2)- Cost‑effectiveness of antenatal telehealth<br>(3)- Satisfaction with antenatal telehealth	(1)- The clinical safety of antenatal telehealth have not found an associated increase in adverse maternal or neonatal outcomes.<br>(2)- Telehealth also seems to be a cost-effective alternative to traditional care models.<br>(3)- Surveys of women and antenatal care providers have shown high satisfaction with telehealth.
**6**	Mei, (2023), USA	Investigate the impact of reduced contact prenatal care by the COVID-19 pandemic on meeting standards of care and perinatal outcomes.	Retrospective case-control study	Reduced contact prenatal care group (**N=90**)<br>Control group (**N=90**)	Telehealth *(Reduced Contact Prenatal Care Model)*	(1)- In reduced contact prenatal care model limiting total number of in-person visits to five for those initiating prenatal care in the model.<br>(2)- All other visits were scheduled as telehealth visits as deemed clinically necessary.	(1)- Primary outcome as standard of prenatal care (e.g. total number of ultrasounds and visits.)<br>(2)- Secondary outcomes included maternal and fetal or neonatal outcomes.<br>(3)- Composite neonatal morbidity.	(1)- There were similar rates of standard prenatal care metrics between groups.<br>(2)- Maternal and neonatal outcomes did not otherwise differ between groups.
**7**	Thirugnanasundralingam, (2023), Australia	Evaluate safety and efficacy of telehealth-integrated antenatal care on pregnancy outcomes in an Australian public health system.	Interrupted time-series analysis	Births in the conventional period (**N=17 873)**<br>Births in the integrated period **(N=8131)**	Telehealth *(Telehealth-integrated Antenatal Care)*	(1)- In conventional care period, women received ten in-person consultations, with additional visits.<br>(2)- Telehealth-integrated antenatal schedule, aiming to deliver 50% of consultations virtually for two models: (Low-risk models were defined as midwifery-led, shared care and High-risk models were obstetric specialist-led care.)	Main outcomes were pre-eclampsia, FGR, and gestational diabetes	(1)- Telehealth-integrated antenatal care replaced around 46% of in-person consultations without compromising pregnancy outcomes.<br>(2)- A reduction in labor induction for suspected FGR, particularly for women in low-risk models, without compromising FGR detection or perinatal morbidity.
**8**	Zhang, (2022), China	Investigate the use of MCH apps among pregnant women in China and explore associations with their outcomes.	Retrospective study	**1393** postnatal women	mHealth *(Different MCH Apps)*	(1)- MCH apps were categorized based on aiming to improve knowledge, behaviors, self-care, and antenatal health services.<br>(2)- The most relevant were health education, counseling, financial transactions and incentives, health status self-monitoring, reminders, appointment making, client -to-client communication, laboratory result checks, diary, games, and hospital service promotion.	Composite adverse pregnancy outcome (CAPO) (Premature birth, low birth weight, birth defects, stillbirth and neonatal asphyxia)	(1)- The most popular app was *Baby Tree* (51.3%).<br>(2)- (62.9%) were intermittent and (37.1%) were continuous users.<br>(3)- The top 3 app features used were health education (100%), health status self-monitoring (54.2%), and antenatal clinic appointment reminders (43.2%).<br>(4)- No statistically significant association was detected between app use and pregnancy outcomes, although the risk of macrosomia seemed to be increased among users who used certain apps.
**9**	Duryea, (2021), USA	Explore the association of audio-only virtual prenatal care with perinatal outcomes.	Cohort study	**6559** women who delivered in 2019, compared with **6048** women who delivered in 2020	Virtual Care/ Visits *(Audio-Only Virtual Prenatal Visits)*	Synchronous audio-only visit types	Composite outcome (two or more component outcomes)	(1)- In the 2020, 67.2% women completed at least 1 audio-only virtual prenatal visit and 20.1% women 3 or more audio-only virtual prenatal visits.<br>(2)- In the 2020 cohort, 173 women (2.9%) experienced the composite outcome, which was not significantly different than the 195 women (3.0%) in 2019 (P = .71).

**10**	Liu, (2021), USA	Identify factors related to satisfaction with virtual visits during pregnancy.	Cross-sectional online survey	**416** pregnant women	Virtual Care/ Visits *(Virtual-Based Prenatal Care)*	Not clearly stated	Patient satisfaction of virtual prenatal care	(1)- 27.9%Women were very or extremely satisfied and 43.5% were moderately satisfied with virtual experiences.<br>(2)- 89.9% of women indicated a preference for in-person care under non-pandemic conditions.<br>(3)- 74.1% of first time moms more preferred in-person prenatal care than 65.2% moms who were pregnant before (p=0.07).
**11**	Peahl, (2021), USA	Evaluate institutional-level adoption, patient and provider experiences with the coronavirus disease 2019 prenatal care model.	Retrospective evaluation study	**253** patients and **77** provider	Virtual Care/ Visits *(Coronavirus disease 2019 prenatal care model)*	Virtual visits scheduled between in-person visits for anticipatory guidance, psychosocial support, and additional services as needed.	(1)- Access (The ability to receive services)<br>(2)- Quality and safety (The ability to deliver medical services, including use of home devices)<br>(3)- Satisfaction (The overall desire to use virtual visits).	(1)- Most patients and almost all providers reported that virtual visits improved access to care (patients, 174 of 253 [68.8%]; providers, 74 of 77 [96.1%]).<br>(2)- More than half of respondents (patients, 124 of 253 [53.3%]; providers, 41 of 77 [62.1%]) believed that virtual visits were safe.<br>(3)- Most reported satisfaction with the coronavirus disease 2019 model (patients, 196 of 253 [77.5%]; providers, 64 of 77 [83.1%]).
**12**	Palmer, (2021), Australia	Assess the effectiveness and safety of telehealth in antenatal care.	Interrupted time-series analysis	**2292** women who gave birth during the telehealth integrated care period.	Telehealth *(Telehealth integrated antenatal care)*	A new integrated antenatal care schedule incorporating telehealth for consultation delivery via voice calls or video calls across all models of pregnancy care.	(1)- Primary outcomes (Detection and management of pre-eclampsia, fetal growth restriction, and gestational diabetes)<br>(2)- Secondary outcomes (Stillbirth, NICU admission, and preterm)	(1)- In low-risk care models women received a mean of five of nine visits by telehealth (56%).<br>(2)- In low-risk care models the number of telehealth missed consultations was higher than in-person consultations.<br>(3)- No significant differences in outcome measures for low-risk care models<br>(4)- Significant reduction in preterm birth among women in high-risk models
**13**	Theiler, (2021), USA	Reduced frequency of in-person prenatal visits by OB Nest program	Using secondary data from the OB Nest randomized, controlled trial	**267** Patient with attrition in the OB Nest arm **(N=131)** and the traditional care arm **(N=130)**	Virtual Care/ Visits *(OB Nest prenatal care model)*	(1)- Eight planned clinic appointments with a physician or midwife.<br>(2)- Six planned virtual (phone or online) connected care visits with a Registered Nurse (RN).<br>(3)- Home digital sphygmomanometer and handheld fetal Doppler.<br>(4)- Access to an online prenatal care community for OB Nest participants.	Cost of care based on OB Nest model compare with traditional care model	(1)- Total provider cost was decreased caring for the OB Nest participants, but nursing cost was increased.<br>(2)- Cost for OB Nest prenatal care was 34% higher than for traditional prenatal care because of additional nursing connected care and etc.<br>(3)- Provider billing increased, travel costs declined, and overhead costs declined in the OB Nest model.
**14**	Tozour, (2021), USA	Evaluate both the patients' and the providers' satisfaction with the administration of maternal-fetal medicine (MFM) services by telemedicine	Cross-sectional survey	**165** patients and **12** provider	Telemedicine *(video visit)*	Telemedicine video visits through the maternal -fetal medicine (MFM)	(1)- Assess the patient's/provider's digital experience<br>(2)- Patients'/ provider's desire for future use	(1)- There were high rates of patient satisfaction in all areas.<br>(2)- They agree that the TM visits were as good as in-person visits (P<.001) and that telehealth made it easier for them to see doctors or specialists (P<.001).<br>(3)- They had higher agreeability scores that telehealth visits saved them traveling time (P=.001).<br>(4)- There was a trend for patients with poor obstetrical history to desire future TM (P=.05).<br>(5)- Provider also demonstrated high levels of satisfaction (83%).

**15**	Barrera, (2021), USA	Perform a literature review of key aspects of prenatal care delivery to inform new guidelines.	Systematic Review	**53** studies	Telemedicine *(The Michigan Plan for Appropriate Tailored Healthcare)*	Home monitoring routine assessments in pregnancy (e.g. maternal blood pressure, fetal heart tones, maternal weight, and fundal height) or frequency of monitoring routine assessments	Maternal and neonatal outcomes, patient satisfaction, and the effect of visit frequency on care coordination	(1)- No differences in maternal and neonatal outcomes among patients without medical conditions with reduced visit frequency schedules.<br>(2)- Home monitoring of blood pressure and weight was feasible, but home monitoring of fetal heart tones and fundal height was not assessed.<br>(3)- Telemedicine was a successful strategy for consultations among individuals with medical conditions; resulted in improved outcomes for patients with depression, diabetes, and hypertension.
**16**	Murthy, (2020), India	Test the mobile phone based voice messaging service in India,(mMitra), would lead to improved antenatal care (ANC) practices, maternal self-care knowledge and health outcomes	Pseudo-randomized controlled trial (single blind)	**2016** pregnant. intervention group **(N=1516)** and control group **(N=500)**	mHealth *[voice message service (mMitra)]*	(1)- One hundred and forty-five audio messages comprised the mMitra call package by BabyCenter.<br>(2)- The audio messages were timed to the gestational age.<br>(3)- The message began with a recognizable 'jingle' to alert the woman and her family; it ended by summarizing the key learning point.	(1)- Primary outcome was anemia reduction.<br>(2)- Maternal health seeking knowledge, attitudes and practices.	(1)- The intervention group performed significantly better than controls on four maternal health practice indicators: receiving the tetanus toxoid ($p = 0.028$), consulting a doctor if spotting or bleeding ($p = 0.025$)<br>(2)- Only one knowledge indicator, on seeking medical care during pregnancy, was statistically increased in the intervention group compared to controls.
**17**	Holcomb, (2020), USA	Evaluate patient satisfaction after integration of audio-only virtual visits into a pre-existing prenatal care schedule	Cross-sectional online survey	From 4,000 audio-only virtual prenatal visits were completed, **283** patients were participate	Virtual Care/ Visits *(Audio-Only Virtual Prenatal Visits)*	(1)- Using online video conferencing sessions, a valid telephone number for confirming virtual visit date and time.<br>(2)- Creating a consistent with previously established guidelines from the WHO & ACOG, including up to four, interspersed synchronous audio-only virtual visits.	Using a four question telephone survey for patient satisfaction, average clinic wait times and attendance rates by visit type	(1)- Nearly 25% of weekly prenatal visits performed through the virtual platform.<br>(2)- On average, 88% of virtual prenatal visits were completed as scheduled, whereas only 82% of in-person visits were attended (P<001).<br>(3)- The majority of patients preferred a combination of in-person and virtual visits for prenatal care.
**18**	Lebrun, (2020), Afghanistan	Assess the feasibility and acceptability of the MAMA pilot program, and to examine changes in reported maternal, (MNCH) knowledge and	Single-group, baseline-follow-up study	**729** women & their husband by voice & text messaging	mHealth *[The Mobile Alliance for Maternal Action (MAMA) program]*	Voice message and SMS text messaging for subscribers at over the 6 month period. Educational messages are sent twice weekly and are timed to the stage of pregnancy	(1)-Overall number of messages received for each participant.<br>(2)- Change in selected MNCH knowledge, attitude and decision making measures between baseline and follow up group.	(1)- Voice message subscribers and female participants more commonly reported missing messages compared with the text message subscribers and men participants.<br>(2)- Over 90% of men and women reported experiencing benefits from the program.<br>(3)- Correct knowledge significantly increased for all (but one MNCH measure at follow up).
**19**	Schramm, (2019), Germany	Elucidate the attitudes of women toward self-monitoring of their pregnancy using noninvasive electronic devices.	A cross-sectional multicenter study	**509** pregnant women (with no previous experience in telemedicine)	Smart device *(Pregnancy Self-monitoring)*	(1)- Patients completed the 21 closed-ended questionnaires on 15 minutes.<br>(2)- The description of the devices in were noninvasive and that women would be able to put them on autonomously.	Attitude toward self-monitoring of pregnancy with smart devices	(1)- Significantly more women regarded it as an alternative prior to seeing a doctor when they perceived a decline in fetal movements (P<.001).<br>(2)- 77.9% (381/489) would like smart wearable devices to measure fetal heart sounds.<br>(3)- The frequency of use of such devices varied from 13.8% (tendency to use several times a day) to 31.6% at most once a week.

**20**	Tobah, (2019), USA	Evaluate the acceptability and effectiveness of OB Nest, a reduced frequency prenatal care model enhanced with remote home monitoring devices and nursing support for low risk pregnant women.	Single center randomized controlled trial	**300** pregnant with attrition OB Nest **(N=134)** Usual Care **(N= 133)**	Virtual Care/ Visits *[OB Nest (OBN) the Mayo Clinic model]*	Participants were assigned to: (1) Eight scheduled clinic appointments, (2) Six virtual (phone or online) connected, (3) Home digital cuff and fetal Doppler, (4) access to an online prenatal care and social network to support with their peers.	(1)-Acceptability was measured by comparing satisfaction with care, perception of stress, and perceived quality of care. (2)-Effectiveness of OB Nest	(1)- Participants in OB Nest had higher satisfaction at 36 weeks (mean group difference [MD] 15.01, 95% CI, 13.38 – 16.64). (2)- Pregnancy related stress was lower in OBN at 14 weeks (MD = -0.09, 95% CI, -0.14 -0.04) and at 36 weeks gestation (MD=-0.06, 95% CI-0.11to -0.01). (3)- Quality of care were not significantly different across arms.
**21**	Marko, (2019), USA	Determine the feasibility of monitoring patients remotely in prenatal care in low-risk pregnancy using a mobile phone app and connected digital devices.	Prospective controlled trial with control group	**88** patients experimental group **(N=47)** control group **(N=41)**	mHealth *(Babyscripts platform)*	Babyscripts app was designed with 2 major goals: (1) To deliver educational content via a mobile App. (2) To remotely monitor blood pressure and weight. Mobile App sends educational content to the expectant mother	(1)- Primary outcome (e.g. number of in-person prenatal care visits) (2)- Secondary outcome (e.g. patient and provider satisfaction with their prenatal care experiences)	(1)- The average number of in-person OB visits during pregnancy was 7.8 and the average number in the control group was 10.2 (P=.01) (2)- There was no statistical difference in patient satisfaction (P>.05) or provider satisfaction (P>.05)
**22**	Masoi, (2019), Tanzania	Test the effectiveness of an interactive mobile messaging alert system on improving knowledge on danger signs, birth preparedness and complication readiness practices among pregnant women	Controlled quasi experimental	**450** women Intervention group **(N=150)** control group **(N=300)**	mHealth *(Interactive mobile messaging alert system)*	(1)- The intervention group was pretested with a baseline questionnaire. Then started receiving and sending messages. They were again given the same questionnaire to ensure consistency as a post-test. (2)- The control group was also pre-tested and received the current standard ANC service. They also completed a post-test after delivery.	(1)- Knowledge on obstetric and newborn danger signs during pregnancy (e.g. vaginal bleeding, swollen hands /face, severe headache, blurred vision, lower abdominal pain). (2)- Individual birth preparedness and complications readiness.	(1)- Mean scores for both knowledge and birth preparedness between the intervention and the control group after the intervention were different (p <.001). (2)- A multivariate linear regression showed a positive association between the intervention (p < 0.001) and level of knowledge (B = 2.910, 95%CI = 2.199–3.621) and birth preparedness (B =1.463, 95%CI = 1.185–1.740).
**23**	Chowdhury, (2019), Bangladesh	An external evaluation of the effect of "*Aponjon*" use on knowledge and behaviors related to maternal and newborn health (MNH) care.	Retrospective observational study	**243** users and **369** non-users for maternal health knowledge and practice indicators	mHealth *[A mobile phone-based service, customized voice messages (Aponjon)]*	(1)- The *"Aponjon"* was to disseminate behavior change communication messages for prenatal care to improve MNH outcomes. (2)- Messages were tailored during their 6–42 weeks of pregnancy. (3)- The *"Aponjon"* system sent two voice messages per week to subscribers, tailored to the timing ANC.	(1)- Knowledge of maternal healthcare. (2)- Behaviors relating to maternal healthcare. (3)- interaction between duration of use of Aponjon services and a measure of 'pattern of receiving and listening to the messages'	(1)- Overall, 84.3% of users said that they had received at least 3 messages per month. (2)- Only about 35.5% women said that they had carefully listened. (3)- 32% of Aponjon subscribers who had received at least 3 messages per month and listened to them were defined as having a 'good pattern of receiving and listening to the messages'. (4)- Did not have an effect on the related outcomes.

**24**	Borsari, (2018), Italia	Testing functionality and acceptability of a new m-Health system in providing ANC care amongst migrants	Pilot feasibility study	**150** migrant pregnant women (from Sicily)	mHealth *[**P**regnancy **a**nd **N**ewborn **D**iagnostic **A**ssessment System (PANDA)]*	With 3 components:<br>(1)-The PANDA app with Android smart-phones provision of standardized ANC by the Community Health Workers (CHWs) through the four modules.<br>(2)- The Point of Care (PoC) is a solar backpack containing a diagnostic platform.<br>(3)-The Medical Unit is a java-based software system inside a referral hospital.	(1)-Functionality by "Red mine" tool: an open source project management web application with a ticket tracking system.<br>(2)-Acceptability by using the actual time spent for each visit.<br>(3)-Overall satisfaction by three questions.	(1)- Almost all tickets (88%) were registered in the first 10 months of the pilot test.<br>(2)- The mean time spent for each visit was 31.9 ± 12.6 min (range 10–58), with significant differences between the mean time of the first visit (mean 40.9 ± 7.9, range 27–58) and the subsequent visits (mean 24.0 ± 10.5, range 10–54).<br>(3)- The overall satisfaction index was > 9 (very satisfied) for 91.9% of women and 7–9 (partially satisfied) for the remaining 8.1%.
**25**	Alhaidari, (2018), Iraq	Determine the feasibility and acceptability of mobile health technology and its potential to improve antenatal care (ANC) services.	A pilot controlled experimental study	**250** women intervention group **(N=100)** for the unexposed (control) group **(N=150)**	mHealth *Text messaging*	A weekly text message was sent to each participant in the intervention group starting from the 6th week of pregnancy.<br>In order to determine the messages sent, client preferences for three types of message themes were explored.	Feasibility and acceptability of m-Health Measuring satisfaction.	There was a statistically significant increase in the median number of antenatal clinic visits from two to four per pregnancy, in addition to being relative of low cost.
**26**	Van Den Heuvel, (2018), Netherlands	Review the current literature on eHealth developments in pregnancy to assess this new generation of perinatal care.	Overview of the Literature	**71** studies That Categorize in 6 domains	eHealth	Studies reporting the use of eHealth during prenatal, perinatal, and postnatal care were included.	Fetal and maternal outcomes	(1)- The use of pregnancy websites and programs for medical information varies from 50% to 98%.<br>(2)- The most searched topics are fetal development, pregnancy complications, healthy lifestyle during pregnancy, general and specific tips / advice during pregnancy and lactation. However, the information on websites is often contradictory and this can lead to confusion.<br>(3)- Remote monitoring and counseling can potentially reduce outpatient visits for antenatal counseling as well as hospitalization for specific clinical reasons.<br>(4)- Users describe patient satisfaction with e-health interventions, describing the high level of comfort and acceptance that leads to greater patient activation and education.
**27**	Barbour, (2017), USA	Analyze patient-related cost and time savings associated with a telemedicine strategy for low-risk prenatal care compared to traditional care.	Secondary analysis of a prospective randomized-controlled trial	**200** women	Telemedicine *(Combination of telemedicine and in-clinic prenatal visits)*	(1)- Telemedicine encounters used a web based platform and patients entered weight, blood pressure, and fetal heart rate into the electronic medical record patient portal.<br>(2)- Time data were collected through self-report at 6 visit time points (20, 24, 28, 30, 34, 36 weeks).	(1)- Primary outcomes (Total visit-related costs and time usage).<br>(2)- Secondary outcomes (Costs and time related to work, personal activities, travel, and childcare).	(1)- The telemedicine care group had significantly fewer in-clinic prenatal visits compared to the traditional prenatal care group (7.2 vs.11.3 visits, p<0.0001).<br>(2)- Women randomized to telemedicine care had a mean of 4.37 (+-1.9) telemedicine visits.<br>(3)- Visit related costs for women in the telemedicine group were significantly lower compared to women in the traditional arm.<br>(4)- Total patient time required for visits was also significantly reduced with telemedicine care (40 minutes per visit and an overall time savings of 3 hours).

**28**	Oliveira-Ciabati, (2017), Brazil	Determine whether PRENACEL SMS service increases the coverage of recommended antenatal care (ANC) practices.	Parallel, cluster-randomized trial	Women from intervention PHCUs groups **(N=770)** (**654** Non-*PRENACEL* and **116** *PRENACEL*) women from control PHCUs group **(N=440)**	mHealth *[Mobile-phone based, short text message service (PRENACEL)]*	(1)- Health care personnel participated PRENACEL workshop.<br>(2)- SMS package consisted of 148 messages (four per week) sending to pregnant women who registered in service.<br>(3)- Women could also send questions, complaints or feedback via SMS free of charge.<br>(4)- All pregnant women attending each groups received routine ANC.	(1)- Primary outcomes (The proportion of women with high ANC Score, a composite measure of coverage of recommended ANC practices)<br>(2)- Secondary outcomes (the coverage of recommended practices)	(1)- The multivariate analysis also suggests that the PRENACEL group (women who read all SMS) had higher mean ANC score [48.5 (±4.2) vs 45.2 (±8.7), p < 0.01].<br>(2)- Higher proportion of women with ≥6 ANC visits (96.9% vs. 84.8%, p = 0.01).<br>(3)- Higher rates of syphilis testing (40.5% vs. 24.8%, p = 0.03) and HIV testing (46.6% vs. 25.7%, p < 0.01) during ANC.
**29**	Feroz, (2017), Pakistan	Assess the effectiveness of m-health solutions on a range of maternal health outcomes by categorizing the interventions according to the types of m-health applications.	Systematic Review	**14** final studies were categorized in to five mHealth applications defined in the conceptual framework.	mHealth	Conceptual Framework on mHealth Applications for Preventive Maternal Healthcare Services	A range of maternal health outcomes.	(1)- In present review, there was five main m-health applications defined which include: 'client education and behavior change communication, registries/ vital event tracking, data collection and reporting, provider to provider communication, and electronic health records.<br>(2)- The most reported use of m-health was for client education and behavior change communication, such as SMS and voice reminders [n = 9, 65%].<br>(3)- Most of the studies showed that m-health interventions have proven to be effective to improve antenatal care and postnatal care services, especially those that are aimed at changing behavior of pregnant women and women in postnatal period.
**30**	Pflugeisen, (2017), USA	Compare the satisfaction of obstetric patients who received one-third of their antenatal visits in videoconference ("Virtual-care") compared to 12–14 face-to-face physician/midwife ("Traditional-care")	Cross-sectional study of previous cohort	Virtual-care **(N = 378)** and Traditional-care **(N = 795)**	Virtual Care/ Visits *[OB CareConnect model (OBCC)]*	Prenatal care is provided to low-risk patients with a mix of five visits conducted in teleconference with an obstetric, Advanced Registered Nurse Practitioner (ARNP) and 7–9 face-to-face physician/midwife visits	(1)- Comparison of patient satisfaction with OB CareConnect prenatal care model with traditional one<br>(2)- Determining the validity and reliability of the questionnaire	(1)- Overall satisfaction was significantly correlated with cohort (F (1,169) = 12.4, p < .001).<br>(2)- Pearson correlations ≥0.4 for domains and their respective questionnaire items and Cronbach's alpha ≥0.7 for each domain.<br>(3)- Virtual-care respondents was not significantly impacted by the incorporation of video-conferencing, Doppler, and blood pressure monitoring technology into their care.
**31**	Pflugeisen, (2016), USA	Implement and evaluate a novel model of prenatal care for low-risk pregnant women that intersperse in-person physician visits	Quality Improvement evaluation study	Virtual-care **(N = 117)** and Traditional-care **(N = 941)**	Virtual Care/ Visits *[OB CareConnect model (OBCC)]*	(1)- Traditional patients had 14 physician visits and a postpartum visit.<br>(2)- Virtual visit had nine physician visits, five prenatal videoconference visits by (ARNP), and a 2-week postpartum videoconference visit.	(1)- Pregnancy and birth outcomes.<br>(2)- Use of the health system.	(1)- A significantly higher percentage of Virtual Visit patients had a preeclampsia diagnosis (p = 0.02)<br>(2)- No other differences were observed between the groups in pregnancy/birth outcomes or health system use

**32**	McNabb, (2016), USA	Assess whether the introduction of the app had an effect on the quality of antenatal care services provided by this lower-level cadre.	Pre/Post-intervention study Single-Group	**266** client exit interviews	mHealth *(CommCare mobile platform)*	(1)- The app dynamically guides CHEWs through antenatal care protocols and collects client data in real time. (2)- Thirteen health education audio clips are also embedded in the app for improving and standardizing client counseling. (3)- To detect changes in quality, they developed an evidence-based quality score consisting of 25 indicators.	(1)- The quality of services rendered, as reported by their clients or indicators. (2)- The level of satisfaction with ANC services received as reported by clients.	The most significant improvements related to health counseling, technical services provided, and quality of health education. (1)- Overall, the quality score increased from 13.33 at baseline to 17.15 at end line ($p<0.0001$), with the most significant improvements related to health counseling. (2)- In terms of technical attributes, the quality score increased from 7.77 at baseline to 8.44 at end line ($p<0.0001$) out of a maximum of 12.
**33**	Lund, (2014), Tanzania (Zanzibar)	Assess antenatal care in a comprehensive way taking into consideration utilization of antenatal care as well as content and timing of interventions during pregnancy.	Cluster randomized controlled trial	**2550** pregnant women intervention group **(N=1311)** and control group **(N=1239)**	mHealth	(1)- Message content was standardized with neutral phrasing and provided as simple text (2)- The messages sent included the following: - A welcome message regardless of gestational age. - Two messages per month before gestational week 36. - Two per week from gestational week 36.	(1)- Four or more antenatal care visits during pregnancy (2)- Tetanus vaccination, preventive treatment for malaria, gestational age at last antenatal care visit, and antepartum referral.	(1)- In the intervention group 44% of the women received four or more antenatal care visits versus 31% in the control group (OR, 2.39; 95% CI, 1.03-5.55). (2)- There was a trend towards improved timing and quality of antenatal care services across all secondary outcome measures although not statistically significant.
**34**	Evans, (2014), USA	Evaluate Text4baby in the military women's population. Its design, methods, baseline (BL) data and outcomes of the program at 4 weeks	Randomized controlled trial	(**459/943**) from enrollees Text4baby group **(N=229)** Control group **(N=230)**	mHealth *[Text-based programs (Text4Baby)]*	(1)- Completing a base line (BL) online survey in the clinic by qualified participant. (2)- Text4baby consists of 135 distinct prenatal text messages delivered on a schedule timed to mothers. (3)- Messages are tied to the information most needed during a particular stage of pregnancy.	Effects of Text4baby on short-term targeted outcomes 4 weeks post enrollment (Nutrition, smoking, taking vitamins, flu shots, alcohol use, health care appointments, health information seeking, and related risk prevention behaviors).	(1)- In the model adjusting, there was a significant effect of Text4baby intervention exposure on increased agreement with belief in the importance of taking prenatal vitamins (OR 1.91, 95% CI 1.08-3.34, P=.024). (2)- In unadjusted models, there were significant effects of intervention exposure on belief in the importance of visiting a health care provider to be a healthy new mother and in the health risks of alcohol during pregnancy ($P\geq.05$).
**35**	Evans, (2012), USA	Assess the efficacy of this text messaging campaign	Randomized pilot evaluation study	**123** women	mHealth *[Text-based programs (Text4Baby)]*	(1)- Exposure group enrolled in the text4-baby message service by staff. (2)- Respondents enrolled in the study completed a 24-item interviewer administered questionnaire. (3)- Text4baby exposure group received standard prenatal counseling and care in addition to the text4baby messages. Control group received only standard prenatal counseling and care. (4)- Participants were interviewed about their knowledge, attitudes, beliefs, and behaviors.	Prenatal care and related health knowledge, attitudes, beliefs, and behavioral outcomes (attending prenatal care visits, nutrition, taking vitamins, getting flu shots, avoiding smoking and related health promoting and risk avoidance behaviors).	Completed 90 follow-up interviews. (1)- A significant effect of text4baby intervention exposure on increased agreement with the attitude statement "I am prepared to be a new mother" (OR = 2.73, CI = 1.04, 7.18, p = 0.042) (2)- A significantly higher overall agreement to attitudes against alcohol consumption during pregnancy (OR = 2.80, CI = 1.13, 6.90, p = 0.026)
