# Supplementary material for: Virtual‐Based Prenatal Care Methods and Their Reported Outcomes—A Scoping Review
Source: Health Sci Rep. 2025 Aug 18;8(8):e71150. doi: 10.1002/hsr2.71150 (PMC12361639; doi:10.1002/hsr2.71150)
Supplement: Supplementary file 2 — New revised appendix 1. [file HSR2-8-e71150-s003.pdf]

Appendix 1

Tables of the search strategy in databases and search engines

Google scholar:

Intitle: prenatal care AND (telemedicine OR telehealth OR virtual care)

search engine	Applied filters	Search strategy	Number reported
Google Scholar Advanced	The word in the title<br>Articles from 2005 to 2021<br>No Citation	allintitle: prenatal care telemedicine OR virtual OR telehealth OR ehealth OR mhealth OR app OR mobile OR digital OR remote	42
	The word in the title<br>Articles from 2005 to 2021<br>No Citation	allintitle: antenatal care telemedicine OR virtual OR telehealth OR ehealth OR mhealth OR app OR mobile OR digital OR remote	70
	The word in the title<br>Articles from 2005 to 2021<br>No Citation	allintitle: obstetric care telemedicine OR virtual OR telehealth OR ehealth OR mhealth OR app OR mobile OR digital OR remote	26
	The word in the title<br>Articles from 2005 to 2021<br>No Citation	allintitle: maternal care telemedicine OR virtual OR telehealth OR ehealth OR mhealth OR app OR mobile OR digital OR remote	75
			213

History and Search Details PubMed

Download 02/01/2021

Search	Query	Results
#3	Search: **#1 AND #2** Filters: **English, from 2005/1/1 - 2021/2/1**	237
#2	Search: **(((prenatal care[Title/Abstract]) OR (antenatal care[Title/Abstract])) OR (maternal care[Title/Abstract])) OR (obstetrics care[Title/Abstract])** Filters: **English, from 2005/1/1 - 2021/2/1**	15,081
#1	Search: **((((((((((((((((((((Telemedicine[Title/Abstract]) OR (Tele-medicine[Title/Abstract])) OR (Mobile Health[Title/Abstract])) OR (mhealth[Title/Abstract])) OR (m-Health[Title/Abstract])) OR (Telehealth[Title/Abstract])) OR (Tele-health[Title/Abstract])) OR (ehealth[Title/Abstract])) OR (e-Health[Title/Abstract])) OR (Remote Consultation[Title/Abstract])) OR (Teleconsultation[Title/Abstract])) OR (Tele-consultation[Title/Abstract])) OR (Telecare[Title/Abstract])) OR (Tele-care[Title/Abstract])) OR (Remote Care[Title/Abstract])) OR (Telemonitoring[Title/Abstract])) OR (Tele-monitoring[Title/Abstract])) OR (Digital Health[Title/Abstract])) OR (Virtual Health[Title/Abstract])) OR (App[Title/Abstract])) OR (Digital app[Title/Abstract])) OR (Mobile app[Title/Abstract])) OR (Virtual Care[Title/Abstract])** Filters: **English, from 2005/1/1 - 2021/2/1**	51,443

Search history Scopus

Download 02/01/2021

History Count	Search Terms	Results
5	((TITLE-ABS-KEY ("prenatal care") OR TITLE-ABS-KEY ("antenatal care") OR TITLE-ABS-KEY ("maternal care") OR TITLE-ABS-KEY ("obstetrics care")) AND PUBYEAR > 2004) AND ((TITLE-ABS-KEY ("Telemedicine") OR TITLE-ABS-KEY ("Tele-medicine") OR TITLE-ABS-KEY ("Mobile Health") OR TITLE-ABS-KEY ("mhealth") OR TITLE-ABS-KEY ("m-health") OR TITLE-ABS-KEY ("Telehealth") OR TITLE-ABS-KEY ("Tele-health") OR TITLE-ABS-KEY ("ehealth") OR TITLE-ABS-KEY ("e-Health") OR TITLE-ABS-KEY ("Remote Consultation") OR TITLE-ABS-KEY ("Teleconsultation") OR TITLE-ABS-KEY ("Tele-consultation") OR TITLE-ABS-KEY ("Telecare") OR TITLE-ABS-KEY ("Tele-care") OR TITLE-ABS-KEY ("Remote Care") OR TITLE-ABS-KEY ("Telemonitoring") OR TITLE-ABS-KEY ("Tele-monitoring") OR TITLE-ABS-KEY ("Digital Health") OR TITLE-ABS-KEY ("Virtual Health") OR TITLE-ABS-KEY ("App") OR TITLE-ABS-KEY ("Digital app") OR TITLE-ABS-KEY ("Mobile app") OR TITLE-ABS-KEY ("Virtual Care")) AND PUBYEAR > 2004) AND (LIMIT-TO (LANGUAGE , "English")) ...View More	543 document results
4	(TITLE-ABS-KEY ("Telemedicine") OR TITLE-ABS-KEY ("Tele-medicine") OR TITLE-ABS-KEY ("Mobile Health") OR TITLE-ABS-KEY ("mhealth") OR TITLE-ABS-KEY ("m-health") OR TITLE-ABS-KEY ("Telehealth") OR TITLE-ABS-KEY ("Tele-health") OR TITLE-ABS-KEY ("ehealth") OR TITLE-ABS-KEY ("e-Health") OR TITLE-ABS-KEY ("Remote Consultation") OR TITLE-ABS-KEY ("Teleconsultation") OR TITLE-ABS-KEY ("Tele-consultation") OR TITLE-ABS-KEY ("Telecare") OR TITLE-ABS-KEY ("Tele-care") OR TITLE-ABS-KEY ("Remote Care") OR TITLE-ABS-KEY ("Telemonitoring") OR TITLE-ABS-KEY ("Tele-monitoring") OR TITLE-ABS-KEY ("Digital Health") OR TITLE-ABS-KEY ("Virtual Health") OR TITLE-ABS-KEY ("App") OR TITLE-ABS-KEY ("Digital app") OR TITLE-ABS-KEY ("Mobile app") OR TITLE-ABS-KEY ("Virtual Care")) AND PUBYEAR > 2004 AND (LIMIT-TO (LANGUAGE , "English")) ...View More	121,280 document results
2	(TITLE-ABS-KEY ("prenatal care") OR TITLE-ABS-KEY ("antenatal care") OR TITLE-ABS-KEY ("maternal care") OR TITLE-ABS-KEY ("obstetrics care")) AND PUBYEAR > 2004 AND (LIMIT-TO (LANGUAGE , "English"))	44,024 document results

Search history ISI

Download 02/01/2021

Set	Results	Save History / Create AlertOpen Saved History
# 3	81	#2 AND #1<br>*Indexes=SCI-EXPANDED, SSCI, A&HCI, CPCI-S, CPCI-SSH, BKCI-S, BKCI-SSH, ESCI, CCR-EXPANDED, IC Timespan=2005-2021*
# 2	5,650	(TI=(Prenatal Care) OR TI=(Antenatal Care) OR TI=(Maternal Care) OR TI=(Obstetrics Care)) *AND* **LANGUAGE:** (English)<br>*Indexes=SCI-EXPANDED, SSCI, A&HCI, CPCI-S, CPCI-SSH, BKCI-S, BKCI-SSH, ESCI, CCR-EXPANDED, IC Timespan=2005-2021*
# 1	35,358	(TI=(Telemedicine) OR TI=(Telemedicine) OR TI=(Mobile Health) OR TI=(mhealth) OR TI=(mHealth) OR TI=(Telehealth) OR TI=(Telehealth) OR TI=(eHealth) OR TI=(ehealth) OR TI=(Remote Consultation) OR TI=(Teleconsultation) OR TI=(Teleconsultation) OR TI=(Telecare) OR TI=(Telecare) OR TI=(Remote Care) OR TI=(Telemonitoring) OR TI=(Tele-Monitoring) OR TI=(Digital Health) OR TI=(Virtual Health) OR TI=(App) OR TI=(Digital app) OR TI=(Mobile app) OR TI=(Virtual Care)) *AND* **LANGUAGE:** (English)<br>*Indexes=SCI-EXPANDED, SSCI, A&HCI, CPCI-S, CPCI-SSH, BKCI-S, BKCI-SSH, ESCI, CCR-EXPANDED, IC Timespan=2005-2021*

Search history ProQuest

Download 02/04/2021

Set	Search	Results
**S6**	(ab(Prenatal Care) OR ab(Antenatal Care) OR ab(Maternal Care) OR ab(Obstetrics Care)) AND ((ab(Telemedicine) OR ab(Tele-medicine) OR ab(Mobile Health) OR ab(mhealth) OR ab(m-health) OR ab(Telehealth) OR ab(Tele-health) OR ab(ehealth) OR ab(e-Health)) OR (ab(Remote Consultation) OR ab(Teleconsultation) OR ab(Tele-consultation) OR ab(Telecare) OR ab(Tele-care) OR ab(Remote Care) OR ab(Telemonitoring) OR ab(Tele-monitoring)) OR (ab(Digital Health) OR ab(Virtual Health) OR ab(App) OR ab(Digital app) OR ab(Mobile app) OR ab(Virtual Care)))	**68**
**S5**	(ab(Telemedicine) OR ab(Tele-medicine) OR ab(Mobile Health) OR ab(mhealth) OR ab(m-health) OR ab(Telehealth) OR ab(Tele-health) OR ab(ehealth) OR ab(e-Health)) OR (ab(Remote Consultation) OR ab(Teleconsultation) OR ab(Tele-consultation) OR ab(Telecare) OR ab(Tele-care) OR ab(Remote Care) OR ab(Telemonitoring) OR ab(Tele-monitoring)) OR (ab(Digital Health) OR ab(Virtual Health) OR ab(App) OR ab(Digital app) OR ab(Mobile app) OR ab(Virtual Care))	**1,989**
**S4**	ab(Prenatal Care) OR ab(Antenatal Care) OR ab(Maternal Care) OR ab(Obstetrics Care)Limits applied	**913**
**S3**	ab(Digital Health) OR ab(Virtual Health) OR ab(App) OR ab(Digital app) OR ab(Mobile app) OR ab(Virtual Care)Limits applied	**1,047**
**S2**	ab(Remote Consultation) OR ab(Teleconsultation) OR ab(Tele-consultation) OR ab(Telecare) OR ab(Tele-care) OR ab(Remote Care) OR ab(Telemonitoring) OR ab(Tele-monitoring)Limits applied	**263**
**S1**	ab(Telemedicine) OR ab(Tele-medicine) OR ab(Mobile Health) OR ab(mhealth) OR ab(m-health) OR ab(Telehealth) OR ab(Tele-health) OR ab(ehealth) OR ab(e-Health)Limits applied	**925**

Summary table of articles searched by different databases/search engine

Originally search result from January 1, 2005, to February 30, 2021		
	**Databases/ Search engine**	**Final Results**
1	PubMed	237
2	Scopus	543
3	Web of Sciences	81
4	ProQuest	68
5	Google Scholar	213
6	SID, Irandoc, and Magiran	6
		1148
Updated records from March 1, 2021, to December 30 2023		
1	From mentioned databases	176
Total		**1324**
